# Posttranslational Modification of the Androgen Receptor in Prostate Cancer

**DOI:** 10.3390/ijms140714833

**Published:** 2013-07-16

**Authors:** Travis van der Steen, Donald J. Tindall, Haojie Huang

**Affiliations:** 1 Department of Urology Research, Mayo Clinic College of Medicine, Rochester, MN 55905, USA; E-Mails: vandersteen.travis@mayo.edu (T.V.S.); tindall@mayo.edu (D.J.T.); 2 Department of Biochemistry and Molecular Biology, Mayo Clinic College of Medicine, Rochester, MN 55905, USA

**Keywords:** androgen receptor, castration-resistant prostate cancer, posttranslational modifications

## Abstract

The androgen receptor (AR) is important in the development of the prostate by regulating transcription, cellular proliferation, and apoptosis. AR undergoes posttranslational modifications that alter its transcription activity, translocation to the nucleus and stability. The posttranslational modifications that regulate these events are of utmost importance to understand the functional role of AR and its activity. The majority of these modifications occur in the activation function-1 (AF1) region of the AR, which contains the transcriptional activation unit 1 (TAU1) and 5 (TAU5). Identification of the modifications that occur to these regions may increase our understanding of AR activation in prostate cancer and the role of AR in the progression from androgen-dependent to castration-resistant prostate cancer (CRPC). Most of the posttranslational modifications identified to date have been determined using the full-length AR in androgen dependent cells. Further investigations into the role of posttranslational modifications in androgen-independent activation of full-length AR and constitutively active splicing variants are warranted, findings from which may provide new therapeutic options for CRPC.

## 1. Introduction

**Figure 1:**
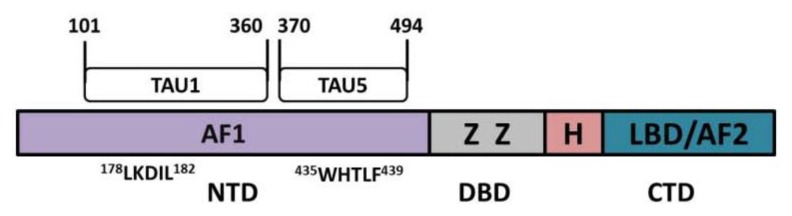
The androgen receptor (AR) contains three major functional domains and several activation functional units that control its activity. The AR protein is comprised of a large NH_2_-terminal domain (NTD), which harbors the transcriptional activation function-1 (AF1), containing the transcriptional activation unit 1 (TAU1) and TAU5. Within the TAU1 and TAU5 regions two core motifs, LKDIL and WHTLF, have been identified to regulate androgen-dependent and androgen-independent AR activity respectively [12,19–22]. Other domains include the DNA binding domain (DBD) that contains two zinc fingers (Z), a short flexible hinge region (H), and the *C*-terminal domain (CDT) that contains the ligand binding domain (LBD) and the transcriptional activation function-2 (AF2).

The androgen receptor (AR) is a member of the steroid hormone receptor family; other family members consist of the estrogen, progesterone, mineralocorticoid, and glucocorticoid receptors [1]. AR plays a vital role in the development of the prostate as well as benign prostate hyperplasia and prostate cancer by regulating cellular proliferation [2–5], survival [6], apoptosis [7] and secretion [2]. AR is a 919-amino-acid protein encoded from a ~180 kb gene that is located at chromosome Xq11-12. AR is comprised of three major functional domains (Figure 1) [8]. The largest, comprising of over half of the receptor, is the *N*-terminal domain (NTD) [9–11], which is highly unstructured and contains one of the two activation function (AF1) motifs. Within AF1 there are two transcriptional activation unit (TAU) regions: TAU1 (residues 101–360) and TAU5 (residues 370–494) [12]. The second functional region in the AR is the DNA binding domain (DBD), which contains two zinc fingers. The first zinc finger interacts with the half-site of the androgen-response element (ARE) [13–15], and the second facilitates dimerization [13,15]. A short flexible peptide sequence called the hinge region connects the DBD to the ligand binding domain (LBD), wherein the second transcriptional activation function (AF2) resides [16–18].

In the absence of androgens, AR is localized primarily in the cytoplasm and remains in an inactive state and interacts with heat shock proteins (HSP90, HSP70, HSP56, and HSP27) [23,24], which prevents it from entering the nucleus [25–27]. Upon binding of androgens to the LBD, AR undergoes a conformational change, which releases bound HSPs; AR dimerizes and is rapidly transported into the nucleus [23,28]. AR dimers interact with the major groove of DNA by binding to ARE sequences. Coactivators and chromatin remodeling complexes are recruited to facilitate transcription of AR target genes [29]. A well-known gene regulated by AR is prostate specific antigen (PSA), which currently is used as a biomarker for prostate cancer (PCa). Besides PSA, AR regulates many other genes that are involved in regulation of proliferation and apoptosis.

**Figure 2:**
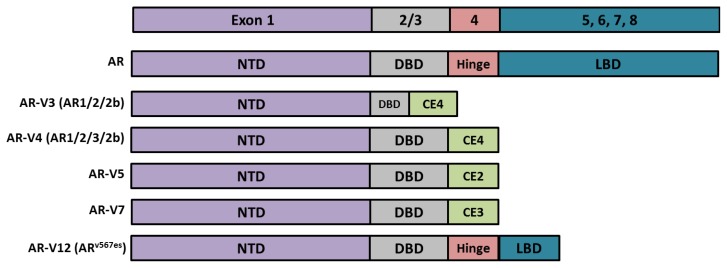
Androgen receptor and its truncated splice variants share structural similarity in the NTD and DBD. Exon skipping or splicing of cryptic exons (CE) yield *C*-terminal truncated AR splice variants (AR-Vs). The splice variants lack the LBD and are constitutively active in the absence of ligand. Increased expression of AR-Vs has been identified in castration-resistant prostate cancer (CRPC).

The role that androgens play in PCa was first described by Huggins and Hodges in 1941 [30], who noted that upon depletion of androgens, prostate tumors shrink. Since then, androgen depravation therapy has been the mainstay of treatment for advanced PCa. Initially, there is a decrease in the mass of the tumor as a result of apoptosis. Unfortunately, PCa usually reoccurs within 18–36 months and becomes a lesion termed as castration-resistant prostate cancer (CRPC) [31–33]. In CRPC, AR becomes activated in the face of castrate levels of androgens. AR expression is often elevated in CRPC [8,34,35], and is believed to be either hypersensitive to androgens [36–38], constitutively active [39], or activated by non-canonical pathways [40]. Moreover, androgens can be synthesized by PCa cells and activate AR in an intracrine fashion [41]. Most recently, constitutively active AR splice variants have been identified [42–47]. These variants are clinically relevant as they are expressed in PCa cell lines, xenografts, and human tumors [45,48,49]. AR-variants (AR-Vs) are not dependent upon androgens for activation as they lack the LBD (Figure 2) [20,50]. AR-Vs contain the AF1 region that encompasses the TAU1 and TAU5 and the DBD. They are found predominantly in the nucleus and drive gene expression constitutively. A comprehensive review of alternatively spliced AR variants was published recently by Dehm and Tindall [51].

AR has two activation function (AF) motifs, AF1 in the NTD and AF2 in the LBD. However, unlike other nuclear receptors where AF2 has strong transcriptional activity, AF1 is responsible for the majority of AR activity [52–54]. In androgen-dependent PCa cells, the activity of the full-length AR depends on ligand binding, where approximately 50% of ligand-dependent activity is mediated by TAU1 [12]. AF1 is also important for the constitutive transcriptional activity of AR-Vs where the LBD is missing. Indeed, the constitutive activity of AF1 in AR-Vs is similar to that of the full-length AR activated by androgens [12]. When the LBD is truncated from the AR, constitutive activity shifts from the TAU1 region to TAU5 [12]. Within TAU5 of the AF1 region is the core sequence ^435^WHTLF^439^ that regulates approximately 50% the androgen-independent activity of AR in CRPC. Mutation of the core motif WXXLF into AXXLF resulted in a decrease in AR activity by half in C4-2 cells while in androgen-dependent LNCaP cells there was no difference in androgen-dependent transcriptional activity of AR [19]. The TAU5-dependent constitutive activity of AR suggests that it is a potential target for treatment of CRPC and therefore is of key interest to understand how the constitutive activation of the AR is regulated.

AR undergoes a number of posttranslational modifications that alter its functional activity including transcriptional activity, stability, and cellular localization. Previous reviews by Gioeli and Paschal [55] have identified several of the modifications; specifically, they describe the kinase responsible for phosphorylation of the AR. Similarly, Coffey and Robson reviewed the posttranslational modifications to the AR highlighting acetylation, methylation, SUMOylation, and ubiquitination along with phosphorylation [56]. Lavery and Bevan reviewed the functional consequences of acetylation to the androgen receptor [57], while Clinckemalie *et al*. reviewed the modifications to the hinge region of the AR [58]. Here we describe the known posttranslational modifications that have been identified to-date. Most of the modifications, identified and reviewed, were discovered in overexpression systems or in androgen dependent cell lines. Here we summarize the modifications, along with the cell line to give clarity to determine if the modification is present and could result in the activation of the AR in CRPC. The identification of different modifications between androgen dependent and CRPC may lead to a better understanding of AR reactivation in CRPC.

## 2. Phosphorylation of the AR

**Figure 3:**
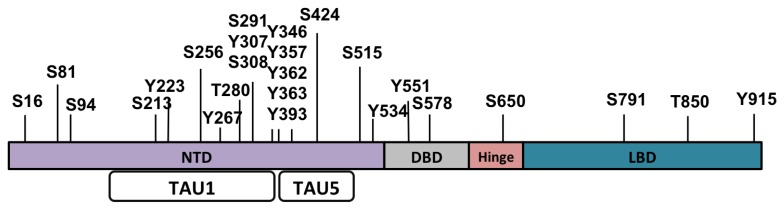
The AR is a phosphoprotein with several serine, threonine, and tyrosine residues that are phosphorylated. The NTD contains most of the phosphorylated residues that regulate AR cellular localization, stability and its transcriptional activity.

The AR is a phosphoprotein with at least sixteen phosphorylated residues identified thus far. Several residues are phosphorylated upon treatment of cells with androgen (testosterone or dihydrotestosterone [DHT]), antiandrogen, or reagents that activate other signaling pathways and alter transcriptional activity, cellular localization, and stability of AR (Table 1). Most of the phosphorylated residues have been identified using the full-length receptor in androgen-dependent cells. It is therefore important to determine in the future, the phosphorylation pattern of the full-length AR in CRPC cells, as well as AR-Vs. The AF1 region has been intensively studied and many of the posttranslational modifications identified have been mapped to this region, specifically the TAU1 and TAU5 regions (Figure 3).

### 2.1. Serine and Threonine Phosphorylation of the AR

Serine 16 (S16) phosphorylation is ligand dependent, as phosphorylation at this site increases upon treatment with ligand [60,77,78]. S16 is a consensus site for both PKA and calcium calmodulin II. However, activation of PKA in LNCaP cells by forskolin, failed to increase S16 phosphorylation as determined by ^32^*P*-phosphopeptide mapping, suggesting that PKA is not responsible for AR S16 phosphorylation. Given that forskolin treatment did increase the overall AR phosphorylation level in LNCaP cells [77], PKA may mediate AR phosphorylation at other site(s). Intriguingly, when the LBD of AR was deleted, it was found that S16 in AR was phosphorylated in the absence of androgens. Thus, it has been speculated that the S16 could be phosphorylated in androgen-independent PCa [78]. This serine residue lies within the region that interacts with the *C*-terminus of AR upon dimerization. Deletion of residues 14–150 impedes the dimerization of AR [79]. Phosphorylation of S16 is androgen dependent in full-length AR, but was phosphorylated in the absence of the LBD, suggesting that this residue may need to be phosphorylated for AR dimerization.

Phosphorylation of serine 81 (S81) is mediated by cyclin dependent kinases (CDKs), including CDK1, CDK5, and CDK9. CDK1, cyclin B1, cyclin B2 and cdc25 are upregulated in PCa [80–85]. Activation of CDK1 by cyclin B resulted in increased phosphorylation of AR [81]. Interestingly, expression of CDK1 increases during the transition from AD to CRPC cells [85]. Cell cycle analysis reveals that there is an increase in AR phosphorylation during mitosis, which coincides with the increased CDK1 activity [64,81]. CDK5 also enhances S81 phosphorylation along with its p35 activator and the p25 byproduct of p35 prevents AR phosphorylation at S81 [59,86]. AR protein degradation is decreased in LNCaP cells overexpressing CDK5 or p35 compared to CDK5 knockdown cells, suggesting that phosphorylation of S81 induces AR stability. Cellular localization is also linked to S81 phosphorylation. Overexpression of CDK5 in LNCaP cells increased AR nuclear localization while knockdown increased AR cytoplasmic localization [59]. CDK9 also regulates the phosphorylation of S81 [59,75,81]. While the pan CDK inhibitor roscovitine blocks the activity of CDK1, CDK5, and CDK9, it inhibits DHT-induced AR phosphorylation at S81. Similarly, when NU6102, the CDK1/CDK2 inhibitor or 3-amino-1*H*-pyrazolo[3,4]quinoxaline, the CDK1/CDK5 inhibitor, were used AR S81 phosphorylation was inhibited [81]. S81 was not highly phosphorylated in LNCaP cells under androgen-depleted conditions, but upon DHT treatment, there was an increase in phosphorylation [74,81]. In contrast, the AR antagonist bicalutamide was unable to increase the phosphorylation at this site. These results were confirmed in CWR22Rv1 and LACP-4 cell lines, suggesting that phosphorylation at S81 is regulated by androgens [81]. Moreover, S81 was also phosphorylated when the LBD-truncated AR was overexpressed in 293 cells [78]. These data suggest that the LBD is dispensable for S81 phosphorylation. Furthermore, one inhibitor of PKC (bisindolylmaleimide) was unable to prevent AR S81 phosphorylation [77]. However, this inhibitor does not block all PKC isoforms such as ζ, ι and μ. Thus, besides the CDKs, PKC is a putative kinase potentially responsible for AR S81 phosphorylation

AR S81 phosphorylation plays an important role in AR transactivation, cellular localization and stability as well as cell proliferation. Ectopic expression of the S81 phosphorylation-resistant mutant S81A failed to activate the ARE4-Luc reporter gene in androgen-treated HeLa and 293T cells [81]. Moreover, overexpression of CDK5 in HEK293 cells resulted in an increase in S81 phosphorylation and PSA expression following androgen treatment; whereas in CDK5-knockdown cells no such increase was observed [59]. These data suggest that CDK5-mediated S81 phosphorylation plays a role in androgen-induced transactivation of the AR.

It was reported that the S81A mutant remained in the cytoplasm, while a phosphomimicking mutant, S81D, localized in the nucleus in the absence of ligand [64]. In LNCaP cells, there was a decrease in AR nuclear localization when CDK5 was knocked down by siRNAs. Similarly, when S81A was expressed, lack of S81 phosphorylation prevented nuclear localization of AR [59]. S81 phosphorylation also regulates cell proliferation. Using 22Rv1 xenograft as a working model, it was found that CDK5 knockdown (shCDK5) cells gave rise to smaller tumors compared to control knockdown (shGFP) cells [59]. Expression of the S81A mutant in LNCaP cells also resulted in a decrease in cell proliferation compared to wild-type AR [59]. The finding that AR-S81A was lost after several passages in LAPC4 cells further supports its role of S81 phosphorylation in cell growth and proliferation [75]. Phosphorylation by CDK5 (and p35) resulted in increased half-life of AR protein. In contrast, knockdown of CDK5 by siRNAs decreased AR half-life, and the proteasome inhibitor MG132 blocked this effect. Furthermore, expression of CDK5 expression stabilizes wild-type AR but not the S81A mutant [59]. These data suggest that CDK5-mediated phosphorylation of S81 regulates the stability of the AR.

Evidence suggests that serine 94 (S94) in AR is constitutively phosphorylated and hormone insensitive [60,74,77,87,88]. It has been shown that the transcriptional activity of AR was not altered when both S94 and S81 were mutated to alanine (A) although the effect of single residue mutation on AR transcriptional activity was not determined [74,88]. The finding that S94 was phosphorylated when the LBD-truncated mutant of AR was expressed in 293 cells supports the notion that S94 is constitutively phosphorylated [78].

Serine 213 (S213) phosphorylation has been studied extensively. To date, AKT [61] and PIM-1 [76,89] have been shown to be responsible for S213 phosphorylation. Mutation of S213 to alanine results in a decrease in overall phosphorylation of AR. Activation of AKT by PI3K increases S213 phosphorylation and the PI3K inhibitor LY294002 suppresses phosphorylation [61]. Treatment of LNCaP, 22Rv1 and LAPC4 cells with the inhibitor isosilybin B suppressed PSA expression [90]. In addition to altering the transcriptional activity of AR, isosilybin B was shown to prevent R1881 (a synthetic androgen)-induced nuclear localization of AR in LNCaP cells and inhibit cell growth. AR protein half-life was reduced approximately by half upon treatment with isosilybin B from 13.8 to 6.8 h, suggesting that phosphorylation of AR at S213 is important for protein stability. The decrease in AR protein was due to the recruitment of the E3 ubiquitination (Ub) ligase Mdm2. The proteasome inhibitor MG132 rescued protein degradation. As demonstrated in both androgen-dependent (LNCaP and LAPC4) and -independent cells (22Rv1) [90], Mdm2 formed a protein complex with both AKT and AR as evident in co-immunoprecipitation (co-IP) assays and this complex was lost when AKT was inactive.

Another kinase that regulates the phosphorylation of S213 is PIM-1 [76,89]. PIM-1 has two distinct isoforms, L (long) and S (short), both of which are able to phosphorylate S213 as determined in LNCaP and COS cells. Even though both PIM isoforms are able to phosphorylate S213, it appears that each has a distinct role in regulation of AR. Overexpression of PIM-1S resulted in a decrease in AR half-life, while expression of PIM-1L was unable to alter the half-life of the protein. Phosphorylation of S213 by PIM-1S resulted in the recruitment of Mdm2 as detected by co-IP, while PIM-1L was unable to be co-immunoprecipitated with Mdm2. A decrease in AR protein was detected in the G2 and M phase of the cell cycle [76]. However, during this period of the cell cycle there is a decrease in the amount of activated AKT protein. This suggests that a second kinase is responsible for maintaining the phosphorylation of AR during the G2 and M phases of the cell cycle. PIM-1S is primarily a nuclear kinase. It can be speculated that PIM-1 may maintain the phosphorylated state of S213 upon translocation into the nucleus and promote the degradation of AR upon entry into the cell cycle in proliferative cells.

AR is also phosphorylated at serine 256 (S256) [60,77,91]. Casein kinase II is the putative kinase responsible for this phosphorylation based upon the consensus sequence defined at this site [77]. Phosphorylation was observed at this residue only in the presence of androgens [60,77]. The function of this residue phosphorylation has yet to be determined.

Two residues in close proximity of each other, threonine 280 and serine 291, are phosphorylated by Aurora A (AurA) in both the absence and presence of androgens. Expression of AurA is elevated in androgen refractory LNCaP derivative cells but not in the androgen sensitive parental LNCaP cells. Furthermore, higher levels of AurA were observed in LAPC-4, 22Rv1, DU-145, and PC-3 cells compared to LNCaP cells. In the absence of androgens, forced expression of AurA increased ARR3-Luc activity in LNCaP cells, while expression of an inactive AurA impeded ARR3-Luc activity. Treatment of LNCaP cells with R1881 had a minimal enhancement of luciferase activity in cells expressed either the active or inactive AurA. This data suggests that T280 and S291 are not critical for androgen induction of AR’s transcriptional activity, but that they may be important in androgen-independent AR activation. These two residues may also work in concert with other phosphorylated residues to enhance AR activity. For example, AurA regulates the activation of cyclin B1/CDK1 [92], which is known to phosphorylate S81 [69].

One residue identified in the TAU1 region to negatively regulate the transcriptional activity of AR is serine 308 (S308) [60,93]. S308 is phosphorylated by cyclin D3/CDK11^p58^, which decreases the AR’s transcriptional activity [93]. Phosphorylation of this residue was identified through mutational analysis of residues in the TAU1 region, and decreased AR phosphorylation was observed upon mutation of S308 to a nonphosphorylatable alanine residue [60]. COS1 and PC-3 cells transfected with AR and CDK11^p58^ and/or cyclin D3 yielded a decrease in luciferase activity (MMTV-Luc) compared to control vector-transfected cells treated with DHT. A similar result was observed in LNCaP cells. An increase in cyclin D3/CDK11^p58^ activity was observed upon DHT treatment of LNCaP cells [93]. Thus, activation of cyclin D3/CDK11^p58^ by DHT induces S308 phosphorylation and decreases AR activity although this effect can be surpassed by other mechanisms, resulting in overall activation of the AR by DHT.

Serine 424 (S424) is a phosphorylation site within the TAU5 region of AR [60,77]. Alteration in S424’s phosphorylated state has yet to be identified. In conjunction with mutation of six additional residues (S16, S81, S94, S256, S308, and S650) there was a decrease in AR activity [60]. Mutational analysis of S424 to alanine resulted in the loss of phosphorylation at this site [77]. However, no studies have identified a role, or the kinase responsible, for S424 phosphorylation. Further studies on this residue are of importance as it is the sole residue that has been identified to date to be phosphorylated within the TAU5 region, which plays a crucial role in androgen-independent activation of the AR.

Serine 515 (S515) undergoes phosphorylation mediated by CDK7 and MAP kinase [70,71]. The kinase CDK7 is part of the TFIIH transcription complex that regulates the two ubiquitination E3 ligases carboxyl-terminus of Hsc70-interacting protein (CHIP) and mouse homologue of double minute 2 protein (Mdm2). A mutant S515A that inhibited phosphorylation was found to co-IP with CHIP but was unable to bind to Mdm2. Both CHIP and Mdm2 appear to be equally associated with AR and both E3 ligases are able to promote AR polyubiquitination while monoubiquitination was only identified when CHIP was present [70]. A phosphomimetic mutant S515E appears to increase the transcriptional activity of AR as measured in a PSA luciferase assay. Also, the phosphorylation state of S515 alters the half-life of AR. The S515 phosphorylated AR or the phosphomimetic mutant S515E had a shorter half-life presumably due to the recruitment of Mdm2 to promote AR for protein degradation since Mdm2 had a lower affinity for binding to the S515A mutant. AR protein degradation could be rescued using the MG132 proteasome inhibitor demonstrating that AR protein loss was a result of degradation [70]. Epidermal growth factor (EGF) treatment induces S515 phosphorylation in COS cells expressing truncated ARΔ507-660. Mutation of S515 to alanine negated AR phosphorylation induced by EGF. Treatment with the MAPK inhibitor U0126 also decreases S515 phosphorylation. As demonstrated in CWR-R1 cells derived from the CRPC cell line CWR22, expression of the phosphorylation-resistant alanine mutation at S515 resulted in decreased AR transcriptional activity as measured by PSA-Enh-Luc assay [71].

Although serine 578 (S578) lies within a PKC consensus phosphorylation motif, the kinase responsible for phosphorylation at this site is still undetermined. Treatment of CWR-R1 and Ishikawa cells with EGF resulted in an increase in AR transcriptional activity, which coincided with an increase in phosphorylation of S578. Both FLAG-AR-(amino acids 507–660)-S578D (phosphomimetic) and FLAG-AR-(amino acids 507–660)-S578A (phosphorylation-resistant) were transfected into COS cells to investigate cellular localization of AR. The phosphomimetic mutant was equally distributed between the cytoplasm and the nucleus, while the alanine mutant exclusively resided in the nucleus [71]. These findings suggest that S578 or other residues in this region (507–660) play an important role in regulation of AR cellular localization.

Phosphorylation of serine 650 (S650) occurs both in the presence and absence of androgens [77] and regulates AR localization and transcriptional activity [73,74,77,87]. In COS-1 cells S650 was constitutively phosphorylated (within 5 min of protein synthesis). However, this was not observed in other cell lines [87]. S650 is present within the CK II kinase phosphorylation consensus motif; but the CK II inhibitor DRB was unable to prevent the phosphorylation of S650 in LNCaP cells [73]. Stimulation of LNCaP cells with an upstream activator of PKC, phorbol-12-myristate-13-acetate, resulted in a phosphorylation event at S650, whereas use of one PKC inhibitor (bisindolylmaleimide) was unable to prevent the phosphorylation event [77]. These observations suggest that the bisindolylmaleimide-insensitive isoforms of PKC (ζ, ι, and μ) may be responsible for S650 phosphorylation. It is also possible that PMA affects AR S650 phosphorylation in a PKC independent fashion. In COS7 cells expression of MAPK kinase (MKK) 4/c-Jun *N*-terminal kinase (JNK)1 or MKK6/p38α increased S650 phosphorylation. A minimal increase in phosphorylation was observed when MAPK/ERK (MEK) was overexpressed; but no decrease in S650 phosphorylation was observed upon inhibition of MEK with UO126. However, inhibition of either p38 (SB203580) or JNK1 (SP600125) significantly decreased the amount of S650 phosphorylation. Additionally, when MKK4 or MKK6 knocked down by siRNAs, there was an increase in the expression of PSA mRNA in the absence or presence of DHT [73]. The phosphor-inhibition mutant S650A decreased AR transcriptional activity by 30% [74]. Thus, S650 regulates AR transcriptional activity. A second function identified for S650 is the regulation of shuttling of the AR between the nucleus and the cytoplasm. When p38α and/or JNK1 activity was inhibited there was a reduction in the amount of AR shuttled from donor to acceptor nuclei (heterokaryon shuttling assay), suggesting that the AR was unable to be transported out of the nucleus in the dephosphorylated state at S650 [73]. S650 is located within the DBD/hinge region; this domain also contains the bipartite nuclear localization signal [94]. This bipartite ^617^RKCYEAGMTLGARKLKK^633^ sequence regulates the localization of AR into the nucleus [95–97] and its close proximity to S650 could prevent AR from localizing/exporting out of the nucleus to the cytoplasm. Interestingly, treatment of LNCaP cells with the PKA activator (forskolin) resulted in increased phosphorylation of S650 in the absence of R1881, with maximal phosphorylation observed at 2 h post-treatment [77]. These data suggest that S650 is potentially regulated in a cAMP dependent mechanism.

Phosphorylation of serine 791 (S791) is similar to S213 in that it is dependent on AKT. The phosphomimetic mutant S791D exhibited reduced binding of ligand, a decrease in nuclear translocation, and reduced transcription of p21^CIP1^ via lack of AR binding to the promoter of p21^CIP1^[62]. Phosphomimetic mutations in both S213 and S791 resulted in an impaired translocation of AR to the nucleus in COS1 cells [62]. AR stability was decreased when S791 was phosphorylated, *i.e.*, the half-life of AR protein decrease (by approximately half) and was restored with the proteasome inhibitor MG132. This suggests that phosphorylation of AR at S791 plays a role in the ubiquitination of AR and its degradation; this residue is the only modified residue within 50 amino acids of the ubiquitinated lysine 845 and 847 [62]. IGF-1 treatment activates PI3K, which can signal through two different kinases AKT or p70S6K. Inhibition of p70S6K with rapamycin was unable to prevent S791 phosphorylation, while the inhibition of AKT by the PI3K inhibitor LY294002 negated this site-specific phosphorylation [61].

Threonine 850 (T850) phosphorylation results in AR stabilization and mediates the low-androgen transcriptional activation of AR, due to recruitment of the E3 ubiquitin ligase RNF6. The phosphorylation of T850 is cycle-cycle dependent with increase phosphorylation during the G2 and M phase, which coincides with increased PIM-1 (S or L) expression. PIM-1L phosphorylates AR at T850, in COS-1 cells that expressed AR, and either the long (1L) or short (1S) isoform of PIM, only upon expression of PIM-1L was phosphorylation observed at T850. AR mutation at T850 resulted in lower AR protein levels during the M phase, these data suggest that phosphorylation at T850 stabilizes AR during the M phase. Upon overexpression of PIM-1L, there was no decrease in AR half-life while the overexpression of the 1S did. This suggests that phosphorylation of T850 protects AR from degradation [76]. Ubiquitination of AR by RNF6 does not degrade AR, but increases its transcriptional activity. Knockdown of RNF6 by shRNA in CWR-R1 and LNCaP cells had reduced AR transcriptional activity measured by ARR2-Luc assay. Residues that were ubiquitinated were identified by MS as K845 and K847, which are two highly conserved residues in AR among species. In human CRPC, RNF6 is up regulated and localized in the nucleus, along with PIM-1L. Knockdown of RNF6 resulted in retarded proliferation in C4-2B and CWR-R1 cells suggesting that it plays a role in proliferation most likely due to the expression levels of AR. Interestingly, the interaction between AR and RNF6 was enhanced upon androgen stimulation, suggesting that low levels of androgen are needed for AR phosphorylation at T850 and its protective activity [76].

### 2.2. Tyrosine Phosphorylation of the AR

Two of the AR tyrosine (Y) residues that are phosphorylated are Y267 and Y363. Both sites are phosphorylated by Ack1, which promotes androgen-independent growth of LNCaP and LAPC4 xenografts in castrated mice. This androgen-independent growth may be a result of the increased recruitment of AR to AREs in the absence of androgens. The role of Ack1 in the proliferation of LNCaP cells in the absence of androgens was further supported by other studies, which showed that the expression of an inactive Ack1 (dAck1) had limited effect on tumor growth, and it was also shown that if the dAck1-expressing cells were exposed to DHT, AR failed to induce PSA expression. These data suggest that Ack1 plays an important role in the activation of AR as a transcription factor in both androgen dependent and independent cells. To confirm that Ack1 is critical for the activity of AR, a constitutively active Ack1 (ca-Ack1) was expressed in LNCaP cells. In the absence of ligand there was an increase in AR binding to the PSA promoter, and when cells were treated with low doses of androgen (levels at which no increase was observed when Ack1 was not constitutively active), there was an increase in PSA expression. This suggests that Ack1 sensitizes PCa cells to low levels of androgens and that Ack1 may facilitate progression from AD to AI phenotype [66]. Anti-androgens had no effect on the increased activation of AR when ca-Ack1 was expressed [65]; further supporting the concept that tyrosine phosphorylation is independent of the LBD. Site mutations conducted on each residue demonstrated that Y363F resulted in a moderate decrease in phosphorylation, while Y267F and Y267F/Y363F mutations abolished Ack1-mediated AR tyrosine phosphorylation. The lack of phosphorylation at these residues resulted in the loss of AR binding to the PSA enhancer region. Inoculation of cells expressing a single Ack1-resistant mutation of AR and constitutively active Ack1 in nude mice gave rise to smaller tumors. The effect on tumor growth was further investigated by the use of an Ack1 inhibitor AIM100. It was found that there was a decrease in cell proliferation as more cells were in the G0/G1 phase and fewer in the S phase compared to mock treated cells. EGF was also unable to up regulate PSA mRNA when cells were treated with the Ack1 inhibitor AIM100 [65]. The activation of Ack1 occurs through signaling implemented by Heregulin (HER2) [65,66] and EGF [65].

Another tyrosine residue in AR phosphorylated is tyrosine 534 (Y534), which is a highly conserved residue in AR among species [66,72]. Receptor for activated C kinase 1 (RACK1) and Src signaling phosphorylate Y534.RACK1, was able to inhibit PSA mRNA expression, and to impede the cellular proliferation that is induced by androgens in PCa cells. This decrease was due to the interaction between Src and RACK1 since dephosphorylated Src was not found in a complex with RACK1 but was bound to AR [98]. Similar to the altered phosphorylation state of Y267 and Y363, EGF was able to induce phosphorylation of AR at Y534. EGF treatment of LNCaP cells that expressed an ARR2-Luc reporter resulted in approximately 4-fold increase in activity. These results were similar to the observed role of EGF in phosphorylation of Y267 and Y363. However, Ack1 does not appear to be involved for phosphorylation of Y534. The nonphosphorylatable mutation of Y534F resulted in the loss of transcriptional activity induced by EGF, although treatment with DHT restored AR activity, but wt-AR was more sensitive to DHT than this mutant. This suggests that constitutive activity was lost, but that DHT stimulated cells are able to overcome the lack of phosphorylation at Y534 to initiate transcription. ChIP analysis revealed that EGF-stimulated Y534 phosphorylation resulted in the recruitment of AR to the PSA promoter but not the enhancer region. Intercellular localization was also altered by mutation of tyrosine to phenylalanine. Y534 phosphorylated AR was located in the nucleus while the phenylalanine mutant was unable to translocate when treated with EFG. Inhibitors of Src were able to impede the localization of wt-AR in the nucleus, which correlated with loss of Y534 phosphorylation [72].

Additional tyrosine residues, identified to be posttranslational modified in hormone-refractory prostate tumor xenografts, are Y223, Y307, Y46, Y357, Y362, Y393, Y551, and Y519 [72]. Upon transformation from a hormone-sensitive to a hormone-refractory xenograft, a significant increase in tyrosine phosphorylation is observed using a pan-phosphotyrosine antibody. Mass spectroscopy confirmed the increase in Tyr phosphorylation and identified the additional tyrosine residues that were modified when AR and SRC were coexpressed, which are: Y223, Y307, Y346, Y357, Y362, Y362/363, Y393, Y551, and Y915. These additional residues are predominantly located within exon 1 of the AR, which is conserved between AR full-length and variants. Four modified residues (Y223, Y307, Y346, and Y357) are located within the TAU1 region, which regulates a significant portion of AR’s functional activity. Their functional role is unknown as of now, but it is feasible to speculate that one residue Y307 is phosphorylated in hormone-refractory xenografts and is adjacent to serine 308 that negatively regulates AR’s transcriptional activity upon phosphorylation. Due to the nature of S308’s ability to inhibit transcriptional activity of AR, it would be feasible to suggest that phosphorylation of Y307 may inhibit phosphorylation at S308 or it may negate the negative regulatory effect of S308 phosphorylation.

Mass spectroscopy identified both Y363 and Y362 were phosphorylated in the hormone-refractory xenograft. A single residue within the TAU5 region Y393 increased in its phosphorylation state upon treatment with SRC kinase. The increase in the SRC kinase activity has been correlated with the progression of PCa from the androgen-dependent state to the hormone refractory state [99,100] increased regulatory role that the TAU5 domain plays in AR’s activity, suggesting that this residue along with the S424 may play key roles the constitutive activity of AR in CRPC.

One tyrosine residue (Y551) is located within the DBD of AR and one in the LBD (Y915) was also identified by mass spectroscopy when SRC was overexpressed. The functional role of these phosphorylated tyrosine residues are unknown, but do require further investigation to determine if these modifications result in the constitutive activity of AR. The increase in SRC activity in CRPC and the increase in Tyr phosphorylation suggest that these are a possible therapeutic option to ablate the activity of AR and slow or inhibit the progression of PCa.

There is an increase in tyrosine phosphorylation in CRPC [101]. In androgen-depleted conditions, tyrosine kinase, non-receptor, 2 (TNK2 or ACK1), SRC, and erythroblastic leukemia viral oncogene homolog 2 (ERBB2 (HER-2/Neu)) tyrosine kinase activity can restore AR function in PCa cells [66,68,72,102]. One of the factors described previously, is the increase in SRC activity. SRC is a downstream kinase from EGF. PTPN11 (protein tyrosine phosphatase) inhibition leads to a decrease in xenograft growth of prostate tumors and reduces the activity of SRC. SRC is also able to interact with the intercellular region of ERBB2 (HER-2), suggesting that SRC may be a key node in the signaling cascade in advanced PCa [101].

Craft *et al*. showed that forced expression of Her-2/neu expression lead to androgen independence of PCa, suggesting that Tyr phosphorylation may play a pivotal role in this transformation [103]. Additionally, AKT pathway is associated with PCa progression and AI tumor growth [104], forced expression of AKT in LNCaP cells accelerated tumor growth [105]. In clinical samples, Her-2/neu was overexpressed in all AI tested, and AKT or ERK1, 2 or both pathways were activated in a majority of AI samples. This suggest that activation of AKT or ERK aids in the escape to hormone refractory PCa. Combined treatment of castration and the tyrosine kinase inhibitor trastuzumab (antibody binds and inhibits Her-2) reduced the risk of recurrence further supporting that tyrosine plays a key role in CRPC, while trastuzumab alone had not effective suppression on tumor growth: Suggesting that it is not only tyrosine phosphorylation but other conditions are needed to allow escape [101].

Phosphorylation of tyrosine (Y) resides in AR have been implicated in the progression to CRPC [101]. Although phosphorylation of tyrosine residues is not usually observed in healthy prostate cells, either due to low phosphorylation levels or because of the short turnover time of the phosphorylation. Phosphorylation of Y534 has been observed in CRPC specimens by immunohistochemistry [72]. Also, several PCa cell lines exhibit increased tyrosine phosphorylation when stimulated by cytokines and other growth factors.

### 2.3. Phosphatases That Affect the AR

The reversible process of phosphorylation of the AR has not been extensively studied. The dephosphorylation of AR occurs through protein phosphatases PP2A and PP1 [60,78,91]. PP2A was found to interact with agonist-bound AR in COS7 and 293T cells. The conformational change that results from androgen binding to AR, allows for the binding of PP2A to the C-terminus of AR [60]. PP2A was only loaded onto the AR when cells expressed the SV40 small t antigen, which resulted in the dephosphorylation of AR at S81, S94, S258, S308, and S424 [60]. Further studies found that the LBD was needed for PP2A binding [78].

Inhibition of PP2 with fostriecin (in the absence of SV40 small t antigen) resulted in an increase in AR protein in LNCaP, HeLa, COS1 and 293T cells [91]. Inhibition of PP1 resulted in an increase phosphorylation of S650 and increased nuclear localization. Modest phosphorylation of AR at S256 and S424 was observed when PP1 was inhibited, suggesting that PP1 plays a critical role in regulating the phosphorylated state of AR [91]. PP1 overexpression resulted in increased AR protein levels and enhanced transcriptional activity. The increased level of protein was a result of the inhibition of the proteasome-mediated AR degradation, suggesting that specific AR phosphorylation is required for AR ubiquitination and degradation. In LNCaP and CWR22Rv1 cells that were treated with the pan phosphatase inhibitor okadaic acid, there was a decrease in endogenous AR protein, and the loss of protein could be rescued by treatment with MG132. Knocking down PP1 with siRNAs in LNCaP and C4-2B cells that were treated with DHT resulted in a decrease in AR expression, further supporting a role for phosphorylation in AR degradation.

## 3. Acetylation of the AR

**Figure 4:**
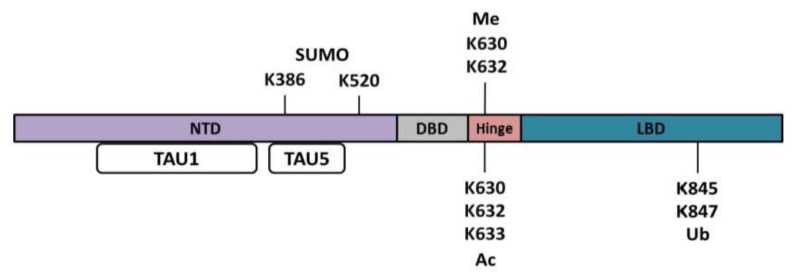
AR transcriptional activity and protein stability are regulated by additional posttranslational modifications to the AR protein. SUMOylation (SUMO) occurs in the NTD, while methylation (Me) and acetylation (Ac) modifies the hinge region and ubiquitination (Ub) takes place in the LBD. These modifications alter transcriptional activity and protein stability.

Acetylation has been found on three residues within the hinge region of the AR. The three residues K630, K632, and K633 are important for the ligand–dependent activation of AR (Figure 4). Mutational analysis of these residues to alanine resulted in no acetylation being detected on the AR, suggesting that these three residues are the major sites of acetylation. Inhibition of the histone deacetylase (HDAC) proteins resulted in an increase in acetylated AR as determined by immunoprecipitation assays using a pan acetylation antibody. The acetylation of AR at residues K632 and K633 are mediated by p300 and p300/cAMP-response element binding protein association factor (p/CAF) [106]. Also, an increase in the amount of acetylated AR was detected upon DHT stimulation as demonstrated by immunoprecipitation using a pan-acetylation antibody. Interestingly, when p300 was overexpressed there was an increase in AR transcription activity (approximately 9 fold over control) while DHT treatment of cells that overexpression of p300 resulted in a reduction in AR transcription activity by nearly half [107]. In PC3 cells cotransfected with AR and a PSA reporter plasmid, while Tip60 was knocked down, there was a decrease in PSA expression. Similarly, in LNCaP cells when Tip60 was knocked down, PSA expression was also suppressed. This decrease in PSA expression and AR activity is likely due to AR localization within the cell as the lysine residues are located within the cellular localization sequence. Fractionation of proteins showed that there was an increase in acetylated AR in the nuclear fraction. Mutation of lysine residues to the non-acetylation-mimic arginine yielded an increase in the cellular localization of AR in the cytoplasm and a decrease in AR transcriptional activity in the hormone-refractory PC3 cell line. The acetylation-mimicking glutamine mutant K630/632/633Q resulted in an increase in nuclear localization and had similar AR transcriptional activity as wild-type AR. Along with decreased AR transcriptional activity, there was a decrease of proliferation in LNCaP, 22Rv1, and VCaP cells when Tip60 was knocked down [108,109]. In support of the suggestion that Tip60 plays a pivotal role in the activation of AR in CRPC, there is an increase in Tip60 expression upon the transformation of LNCaP cell to the LNCaP derived CxR cell line (CRPC).

Deacetylation of AR can be mediated by HDAC1. Inhibition of HDAC1 resulted in an increase in the amount of acetylated AR protein, which had increased activity in response to low concentrations of androgens [107]. A second AR deacetylase that suppresses its transcriptional activity is SIRT1, a nuclear NAD-dependent deacetylase [110–112]. Deacetylation of AR by SIRT1 was identified by mass spectroscopy upon treatment with androgen [112]. Overexpression of SIRT1 in LNCaP cells and coexpression of PSA-Luc resulted in a 3-fold reduction in AR activity upon DHT stimulation. Upon depletion of SIRT1 by RNAi and DHT treatment there was a 3-fold increase in PSA-Luc. Upon depletion of SIRT1 by RNAi and DHT stimulation there was a 2.5-fold increase in AR activity compared to control. Similar results were obtained upon inhibition of SIRT1 by NAM resulted in an increase of PSA and KLK2 transcripts. Activation of SIRT1 by resveratrol resulted in a suppression of both transcripts [110]. Similar results were obtained in spinal and bulbar muscular atrophy (SBMA) where AR is modified by SIRT1. In deacetylated AR, there was a decrease in AR transcriptional activity demonstrating that SIRT1 deacetylation of AR is not PCa specific [111].

## 4. Methylation of the AR

Similar to histones and other proteins, AR can be methylated. Methylation of AR occurs at two residues within the hinge region. These two residues, lysine 630 and 632, overlap with the acetylation residues (Figure 4). Both of these modifications occur to the residues that are part of AR’s nuclear localization signal. When wt-AR and K630A (mutation unable to be methylated or acetylated) were expressed in COS cells, there was no change in the transcriptional activity of AR. This mutation also blocks the potential for acetylation, suggesting that in its non-methylated and non-acetylated state AR transcriptional activity is unaltered. Knockdown of Set9, the methyltransferase responsible for AR methylation inhibits AR binding to the promoter of PSA in LNCaP cells while overexpression of Set9 enhances AR engagement in the PSA promoter. The lysine demethylase family (KDM) are coactivators of AR, the family is comprised of KDM4A, KDM4D [113], KDM4C [114], and KDM4B [115]. The knockdown expression of KDM4B in LNCaP cells resulted in a decrease in AR activity (by 38% for PSA), while knockdown of the other family members did not alter AR function. Validation that KDM4B was a regulator of AR activity, expression of KLK2, TMPRESS2, NKX3.1, and N-myc downstream-regulated 1 (NDRG1) also were down regulated when KDM4b was suppressed. Co-immunoprecipitation determined that AR and KDM4B interact within 30 min of DHT treatment (an enrichment of 2.7 fold), but after 2h the interaction was lost [115]. This suggests that non-activated AR is methylated and upon DHT stimulation, AR is demethylated which may allow for K630 and K632 residues to be acetylated. In addition to the decrease in AR methylation, upon knockdown of KDM4B there was a decrease in H3K9 acetylation, suggesting that KDM4B directly demethylates histones or that due to the methylation state of AR, recruitment of necessary histone demethylases is disrupted.

## 5. Ubiquitination of the AR

Ubiquitination (Ub) is a small covalent modification that has been identified on two lysine residues in the LBD of the AR (Figure 4). Ubiquitination of lysine 845 (K845) and K847 were identified by mass spectrometry [116] and these modifications are mediated by RNF6. RNF6 promotes AR monoubiquitination and enhances transcriptional activity of AR by polyubiquitination. In CWR-R1 and LNCaP cells treated with androgens, there was an increase in global ubiquitination of the AR. Upon knockdown of RNF6 by siRNA, there was a decrease in AR transcriptional activity measured by luciferase assay [116]. In contrast, polyubiquitination of the AR can be catalyzed by the E3 ligases MDM2 and CHIP [116–119]. MDM2 and CHIP-mediated polyubiquitination of the AR promotes AR proteasome degradation [117–119].

AR’s increase in expression and/or stability is hypothesized as being a possible mechanism that can sensitize androgen receptor to low levels of androgens, and possibly lead to castration resistance. One mechanism that could result in the increase in AR protein is the lack of ubiquitination and degradation of AR. A second possibility that could reduce AR’s transcriptional activity is AR’s cellular localization, which is regulated by its nuclear export signal (NES^AR^). The NES^AR^ is located within the LBD (between residues 743–817) and is only found in the full-length and is absent in the variants [120]. The NES^AR^ fusion protein had a decrease in half-life compared to GFP-tagged protein. There are no known residues that are ubiquitinated in AR within this sequence; however, these data suggest that this is a potential degron sequence. When GFP-NES^AR^ protein was expressed in LNCaP or C4-2 cells, it had a shorter half-life compared to GFP protein alone. Treatment with MG132 rescued the loss of GFP-NES^AR^ while it had no effect on the GFP alone in transfected cells. In PC3 cells transfected with His-tagged ubiquitin and GFP or GFP-NES^AR^, there was an increase in ubiquitinated GFP-NES^AR^ fused protein. Interestingly, inhibition of the proteasome resulted in the aggregation of the NES^AR^ in aggresomes. The two lysine residues that are known to be ubiquitinated are located downstream of the NES and only found in the full-length AR. The aggregation of the NESAR into aggresomes is a possible mechanism of protein degradation for the AR-variants as they lack the LBD and the residues that are known to be ubiquitinated [121].

The ubiquitination status of AR alters its transcriptional activity and localization. The ZIPK (zipper-interacting protein kinase) is a coactivator of AR, and upon knockdown by siRNA, there is an accumulation of AR at AREs, and decreased polyubiquitination. Knockdown of ZIPK reduces mRNA expression by 20%–40% due to the recruitment of AR to the promoter and enhancer regions of PSA and KLK2. Within 90 min of DHT treatment, ZIPK interacts with AR while in the second cycle (>180 min) there was no ZIPK. Knockdown of ZIPK yields similar results as inhibition of proteasome by MG132 rescues AR protein. Coexpression of ZIPK and Mdm2 in HEK293 cells yields enhanced luciferase activity, while coexpression of Mdm2 and a dead ZIPK (ZIPKD161A) had a decrease in Mdm2 phosphorylation. DHT treatment increased the amount of monoubiquitnation, while coexpression of Mdm2 enhanced polyubiquitination of AR; similarly, coexpression of ZIPK also resulted in an increase in polyubiquitination. The coexpression of the ZIPKD interfered with the increase of polyubiquitination and reintroduction of the ZIPK rescued AR polyubiquitination [122].

## 6. SUMOylation of the AR

AR can also undergo SUMOylation, which is the attachment of approximately 100 amino acids of small ubiquitin-like modifier (SUMO) to lysine residues [123]. SUMOylation alters the regulation of transcription, cell cycle, nucleocytoplasmic transport, DNA replication and repair, and apoptosis. Unlike ubiquitination, SUMO does not depend upon the linkage of SUMO residues to bestow information. SUMO is a reversible covalent isopeptide linkage modification that can regulate or alter the cellular targets of proteins [124]. Similar to the ubiquitin pathway, SUMOylation involves three different catalytic enzymes (E1, E2, and E3). The E3 ligases that are found responsible for AR SUMOylation include PIAS1 and PIASx-alpha [125,126] (35). Only a small fraction of AR protein is SUMOylated within the cell [127]. The two SUMOylation sites in AR, lysine 386 (K386) and 520 (K520) (Figure 4), have so far been identified [128] (13, 32). The SUMOylation enzymes E1 and E2 are highly concentrated in the nucleus, and it is suggested that AR is SUMOylated upon transport into the nucleus. However, SUMOylated AR is found in both the cytoplasm and nucleus. Mutational analysis of AR for K386R alone or in combination with K520R resulted in a 2 to 3-fold enhancement of androgen-dependent transcription in promoters containing AREs [97,127,129] (13, 32), suggesting that SUMOylation plays an important role in regulation of AR activity. In VCaP cells, SUMO-2/3 (both SUMO2 and 3 are nearly identical) is modified due to cellular stress (temperature change). AR is also dissociated from chromatin resulting in a decrease in activity, but remains in the nucleus. Disruption of AR’s SUMO sites (double mutant) had a small but significant retarding effect on mobility of AR in the presence of androgen. Compared to previously reported data for SUMO-1 altering the activity of AR, when SUMO-2 is overexpressed there was a 7-fold increase of PSA mRNA compared to SUMO-1. AR-SUMO regulates gene expression in HEK293 cells: either AR-wt (SUMOylated) or arginine mutant had a decrease in AR binding to FOXO4 binding, while SPOCK1 had an increase in expression. Interestingly, AR is modified with SUMO-2 in the presence of androgen (testosterone) but not when treated with antiandorgen (Bicalutamide), and SUMO-1 was not found to be covalently linked to AR in VCaP cells. Site mutation of lysine K386 and/or K520 to arginine to prevent SUMO binding resulted in no SUMO-1 and a small amount of SUMO-2/3 bound to AR. These data suggest that there are additional AR residues that are SUMOylated. Androgen treatment is known to modify AR’s stability, resulting in an increase in protein half-life. Mutation of lysine to arginine that prevents SUMO at K386 and K520 resulted in a decreased half-life from 14 to 6 h; and in the presence of androgen there was an increase in life span to 22 and 14 h respectively [130].

## 7. Conclusions

The posttranslational regulation of the AR has been extensively studied in androgen dependent cells. However, there have been few studies that have investigated the role of these modifications in CRPC. The activation of AR in androgen dependent cells is well established upon androgen treatment. However, the question of how AR is constitutively activated in CRPC is not well understood. In addition to the activation of the full-length receptor in CRPC, there are several splice variants that are constitutively activated. The reviewed posttranslational modifications to AR described above are mostly completed using full-length AR that was either expressed in cells that do not endogenously express AR (COS, HEK293T, 293 cells), or in androgen dependent cell lines (LNCaP). The identification of these modifications in androgen dependent PCa cells should also give insight into the potential role of these modifications in CRPC.

The major posttranslational modification of the AR is phosphorylation, which regulates AR cellular localization, transcriptional activity, and stability. Since the majority of the modifications are known to enhance transcriptional activation, it would suggest that in CRPC cells, AR might also be phosphorylated at these sites, thereby promoting its constitutive activity. In androgen dependent cells, it was shown that S308 negatively regulates the activity of AR. This residue is located within the TAU1 region, which plays a pivotal role in regulation of androgen independent activation of the AR. In CRPC, AR regulation shifts from the TAU1 to the TAU5 region. Thus, it is important to determine in the future whether S308 phosphorylation plays a leverage role in this transition. Additionally, several residues are phosphorylated upon androgen treatment (S81, S308, and S424) and were phosphorylated upon deletion of the LBD. These data suggest that there are additional mechanisms mediating AR phosphorylation, which may be responsible for AR’s constitutive activity.

The complexity of cross talk and cellular signaling may help explain the constitutive activity of AR. The PI3K inhibitor, PTEN, has been shown to be down regulated or lost in CRPC, which would allow for activation of PI3K and ultimately its downstream effector, AKT. The increase in cytokines (IL-9, Il-6, and Il-4) has the potential to activate signaling pathways that may stimulate the activity of AR by regulating its phosphorylation [131–133]. Several external factors may play a role in the activation of AR. Here we only review the modifications that are shown to directly modify AR.

The AR-variants lack the LBD and are constitutively active. It would be important to define the phosphorylation spectrum of the AR-Vs under androgen deprivation conditions. Findings from this investigation should shed new light on our understanding of the mechanism underlying constitutive activation of the AR and, thereby, provide new avenues for better targeting aberrant activation of the AR in CRPC.

## Figures and Tables

**Table 1:** Phosphorylated residues of the AR and their functional roles.

Residue	Kinase/phosphatase	Function	References
S81	CDK1, CDK5, CDK9	Localization protein stability	[59]
	PP2	Cell growth transcription	[60]

S94	PP2	Transcription	[60]

S213	PI3K/AKT1	Localization	[61–63]
	PIM-1	Stability	[59,64]

Y267	Ack	Cell growth transcription	[65–68]
	Src		

T280/S291	AurA	Cell growth transcription	[69]

S308	PP2	Transcription	[60]

Y363	Ack	Cell growth transcription	[66]

S424	PP2	Transcription stability	[60]
	PP1		

S515	MAPK	Transcription degradation	[70,71]
	CDK7		

Y534	Src	Localization cell cycle transcription	[68,72]

S578		Localization transcription	[71]

S650	ERK1/JNK1/p38-alpha	Localization	[73]
		Transcription	[74]
	PP1	Localization	[75]

S791	PI3K/AKT1	Transcription apoptosis localization	[61–63]

T850	PIM-1L	Stability	[76]
